# Pulsed Electric Fields Alter Expression of NF-κB Promoter-Controlled Gene

**DOI:** 10.3390/ijms23010451

**Published:** 2021-12-31

**Authors:** Justina Kavaliauskaitė, Auksė Kazlauskaitė, Juozas Rimantas Lazutka, Gatis Mozolevskis, Arūnas Stirkė

**Affiliations:** 1 Laboratory of Bioelectrics, Center for Physical Sciences and Technology, Sauletekio Ave. 3, LT-10257 Vilnius, Lithuania; justina.kavaliauskaite@ftmc.lt (J.K.); aukseka2010@gmail.com (A.K.); 2 Department of Botany and Genetics, Institute of Biosciences, Life Sciences Center, Vilnius University, Sauletekio Ave. 7, LT-10222 Vilnius, Lithuania; juozas.lazutka@gf.vu.lt; 3 Laboratory of Prototyping of Electronic and Photonic Devices, Institute of Solid State Physics, University of Latvia, Kengaraga Str. 8, LV-1063 Riga, Latvia; gatis.mozolevskis@cfi.lu.lv

**Keywords:** microsecond pulsed electric field, inducible gene transcription control, reporter assay, secreted alkaline phosphatase, mammalian cells, cell line, NF-κB

## Abstract

The possibility to artificially adjust and fine-tune gene expression is one of the key milestones in bioengineering, synthetic biology, and advanced medicine. Since the effects of proteins or other transgene products depend on the dosage, controlled gene expression is required for any applications, where even slight fluctuations of the transgene product impact its function or other critical cell parameters. In this context, physical techniques demonstrate optimistic perspectives, and pulsed electric field technology is a potential candidate for a noninvasive, biophysical gene regulator, exploiting an easily adjustable pulse generating device. We exposed mammalian cells, transfected with a NF-κB pathway-controlled transcription system, to a range of microsecond-duration pulsed electric field parameters. To prevent toxicity, we used protocols that would generate relatively mild physical stimulation. The present study, for the first time, proves the principle that microsecond-duration pulsed electric fields can alter single-gene expression in plasmid context in mammalian cells without significant damage to cell integrity or viability. Gene expression might be upregulated or downregulated depending on the cell line and parameters applied. This noninvasive, ligand-, cofactor-, nanoparticle-free approach enables easily controlled direct electrostimulation of the construct carrying the gene of interest; the discovery may contribute towards the path of simplification of the complexity of physical systems in gene regulation and create further synergies between electronics, synthetic biology, and medicine.

## 1. Introduction

The possibility to artificially adjust and fine-tune gene expression is one of the key milestones in contemporary bioengineering and synthetic biology [1,2]. In addition, gene expression control has become highly relevant for clinical applications with the rise of advanced targeted medicines such as cell-based therapies, protein drugs, or gene therapies [3,4,5]. Techniques of targeted therapeutics may provide treatment options that are beyond the reach of conventional small-molecule medicines [6,7,8]. However, as with any other therapeutic molecule, effects of proteins or other therapeutic transgene products highly depend on the dosage; therefore, both for bioengineering and for potential therapeutic applications, controlled and regulated gene expression is a requirement [2,9]. Uncontrolled and leaky expression of transfected genetic construct, in some cases, may result in cellular toxicity or stochastic events; for example, therapeutic proteins usually display a narrow safety window, and even slight fluctuations in their amounts might significantly impact function and, subsequently, cell growth or viability parameters [10]. Thus, control over the level of gene expression in a time-dependent and, preferably, reversible manner is an important output in biology as well as innovative medicine.

Early transcription-based inducible gene expression switches exploited endogenous control mechanisms that respond to exogenous signals or stress, for example, cytokines, hormones, heat, metal ions, and hypoxia, but limitations, such as pleiotropy, restrain these strategies from therapeutic applications [11]. Synthetic gene expression switches with electronics-inspired basic circuit elements of input, processing, and output ushered a new era of cell engineering that started changing the scene of biomolecule production, cell reprograming, and novel therapeutics [12]. Synthetic switch approaches operate via heterologous chimeric systems and chemically inducible promoters [13], for example, dimerizer-regulated systems that employ non-immunosuppressive rapamycin analogs [14], the tetracycline (TetR)-inducible systems [15,16,17], or progesterone receptor/mifepristone (RU486)-inducible systems [18,19,20]. These systems are based on chimeric transcription factors and artificial promoters, and they respond to small molecular weight inducers. Despite the encouraging results, the specifics of small molecule activators—such as unfavorable pharmacokinetic properties, adverse effects, or system immunogenicity—hamper chemically induced gene expression systems in therapy, and mentioned issues still need to be addressed [21].

Since endogenous or chemical input might be limited, physical techniques, such as optogenetics [22,23], heat stimulation [24,25], magnetic fields [26], or remote electrical stimulation [27] stepped in and demonstrated optimistic perspectives for transgene expression control. Such approaches may overcome side effects of chemical agents; avoid issues related to bioavailability or pharmacokinetics and pharmacodynamics [28]; and are noninvasive or, in some cases, even traceless. Still, complex sophisticated devices, synthetic biology-driven designer cell biocomputers, and further extensive studies are needed before the physical technology-based gene regulation is widely adopted in a clinical setting of advanced therapies. In this context, pulsed electric field technology could be another candidate for a noninvasive, biophysical regulator of gene expression. Pulsed electric field technology exploits a relatively simple and easily adjustable pulse generating device for direct stimulation of cells, therefore avoiding exogenous small molecules and, in some cases, heterologous genetic elements [29,30]. Successful implementation of pulsed electric fields in gene switch techniques would create a pathway towards the simplification of processes for biotechnological or therapeutic applications; create an additional crosstalk; and interconnect biological circuits with physics, electronics, and synthetic biology.

Over the last few decades, pulsed electric field technology achieved a significant role as both a stimulator and therapeutic modality in biology, biotechnology, and medicine [31,32,33,34,35]. Life sciences predominantly recognize electric fields for the phenomenon termed electroporation [36] and for their ability to aid and deliver nucleic acids, proteins, and other membrane-impermeable molecules into the cell [37,38]. However, accumulated evidence suggests that depending on applied parameters, such as pulse duration and electric field strength, more diverse intracellular effects may occur [39,40]. Cell exposure to pulsed electric fields of ultra-short duration (within the range of nanoseconds) and high voltage (up to thousand V/cm) instigates caspase activation and programmed cell death (apoptosis) [41], activates signaling pathways [42,43], and generates reactive oxygen species and oxidative stress [44]; whereas, several studies have demonstrated that exposure to longer (millisecond scale) and lower voltage (up to 200 V/cm) electrical pulses alter gene expression in mammalian cells [27,29]. This means that pulsed electric fields might indeed behave as noninvasive, nonligand regulators of intracellular processes, including expression of genes; nevertheless, with regards to various pulsed electric field parameters (for example, nano- and microsecond-duration pulses), we still lack experimental data and substantial knowledge to support this assumption.

Such a background prompted us to investigate microsecond-duration pulsed electric fields as potential nonligand physically triggered modulators of gene expression. To address this possibility, we transfected five different mammalian cell lines with a nuclear factor κB (NF-κB) pathway-controlled transcription system, coding a secreted embryonic alkaline phosphatase (SEAP) protein under the control of inducible promoter carrying NF-κB recognition sites. Upon induction of the NF-κB promoter, SEAP secretes into the growth medium of cells, where its activity can be detected by conducting an enzymatic reaction and a colorimetric analysis. We later exposed cells to a range of diverse microsecond-duration pulsed electric field parameters. To prevent cell toxicity (such as irreversible permeabilization and loss of viability), we only took into consideration protocols that would allow the generation of relatively mild physical stimulation [45,46]. Here, we report the response pattern of the NF-κB transcription system to microsecond-duration pulsed electric fields stimulation in tumor (human cervix carcinoma (Hep-2c); human bone osteosarcoma (U-2 OS)), and nontumor (human embryonic kidney (HEK-293); Chinese hamster ovary (CHO-K1); and mouse subcutaneous connective tissue (L-929)) cell lines. To our knowledge, this is the first experimental study and the first proof of principle that microsecond-duration pulsed electric fields can modulate gene expression from transfected DNA constructs in a mammalian cell context.

## 2. Results

### 2.1. Validation of Experimental Setup

**Figure 1:**
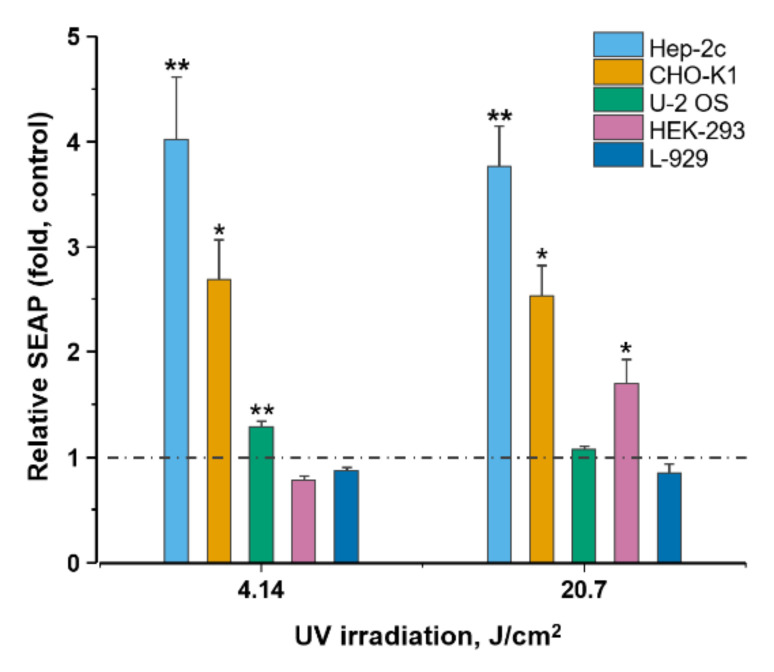
NF-κB/SEAP response system performance in different mammalian cell lines after UV irradiation. Indicated cell lines were transfected with a NF-κB-driven SEAP expression plasmid (pNF-κB-SEAP) and exposed to low (4.14 J/cm^2^) or high (20.7 J/cm^2^) UV doses 24 h before SEAP expression was quantified in the culture supernatant. Dashed line denotes baseline of negative control (cells cultivated under otherwise identical conditions, but in the absence of UV). Significances compared with control were determined using student’s *t*-test: * *p* < 0.05, ** *p* < 0.01.

To evaluate if the activity of the response system can be changed by the application of physical stimuli, we first tested the activity of the NF-κB/SEAP reporter system after stimulation with a well-known physical agonist of the NF-κB pathway—UV light (Figure 1). Obtained results indicate that UV light induced the NF-κB/SEAP reporter system in Hep-2c and CHO-K1 cells. In the Hep-2c cell line, the reporter system activity was approximately 4 times (4.02 ± 0.59, *p* < 0.01) higher at 4.14 J/cm^2^ UV dose and approximately 3.8-fold (3.76 ± 0.38, *p* < 0.01) higher at 20.7 J/cm^2^ compared with negative control. The system expression was approximately 2.7-fold (2.70 ± 0.38, *p* < 0.05) higher in CHO-K1 cell line when 4.14 J/cm^2^ UV dose was applied and approximately 2.5-fold (2.53 ± 0.29, *p* < 0.05) higher at the dose of 20.7 J/cm^2^. The higher UV dose had a negligible effect on the reporter system expression in U-2 OS cell line; however, the lower dose caused approximately 1.3-fold (1.28 ± 0.06, *p* < 0.01) higher expression rate compared with the control. By contrast, we observed no increase in the expression of the reporter system at the lower UV dose in HEK-293 cell line, while the higher dose resulted in approximately 1.7-fold (1.70 ± 0.23, *p* < 0.05) increase compared with the control. UV doses of 4.14 J/cm^2^ and 20.7 J/cm^2^ resulted a slight decrease in reporter system activity (0.87 ± 0.03 and 0.85 ± 0.08, respectively, *p* > 0.05) in L-929 cell line. To evaluate if further changes in SEAP expression levels could be observed at even higher UV irradiation doses, we exposed L-929 cells to UV of 41.7 J/cm^2^. The results indicated further decrease of expression levels of the reporter system (0.58 ± 0.06, *p* < 0.001, not shown). The data show different NF-ĸB/SEAP expression dynamics among cell lines, but overall, it passed the validation test for a physical stimulus response model except for L-929 cells. Nonetheless, we decided to investigate if L-929 can yield different result in response to microsecond-duration pulsed electric fields.

### 2.2. Membrane Permeability after Microsecond-Duration Pulsed Electric Fields Treatment

**Figure 2:**
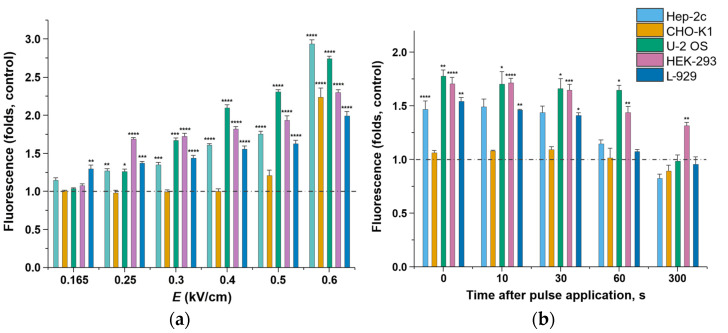
(**a**) Membrane permeabilization after microsecond-duration pulsed electric fields treatment at the field strengths of 0.165, 0.25, 0.3, 0.4, 0.5, and 0.6 kV/cm. Eight square wave pulses of 100 µs-duration and 1 Hz-frequency were applied. SYTOX^®^-green stain uptake was measured one hour after pulsed electric field treatment. The dashed line denotes baseline of negative control (cells cultivated under otherwise identical conditions, but in the absence of pulsed electric fields). (**b**) Membrane reseal assay at *E* = 0.4 kV/cm. SYTOX^®^-green stain was added 0, 10, 30, 60, and 300 s after pulsed electric field treatment; fluorescence was read one hour later. Result significances compared with control were determined using student’s *t*-test: * *p* < 0.05, ** *p* < 0.01, *** *p* < 0.001, **** *p* < 0.0001.

**Figure 3:**
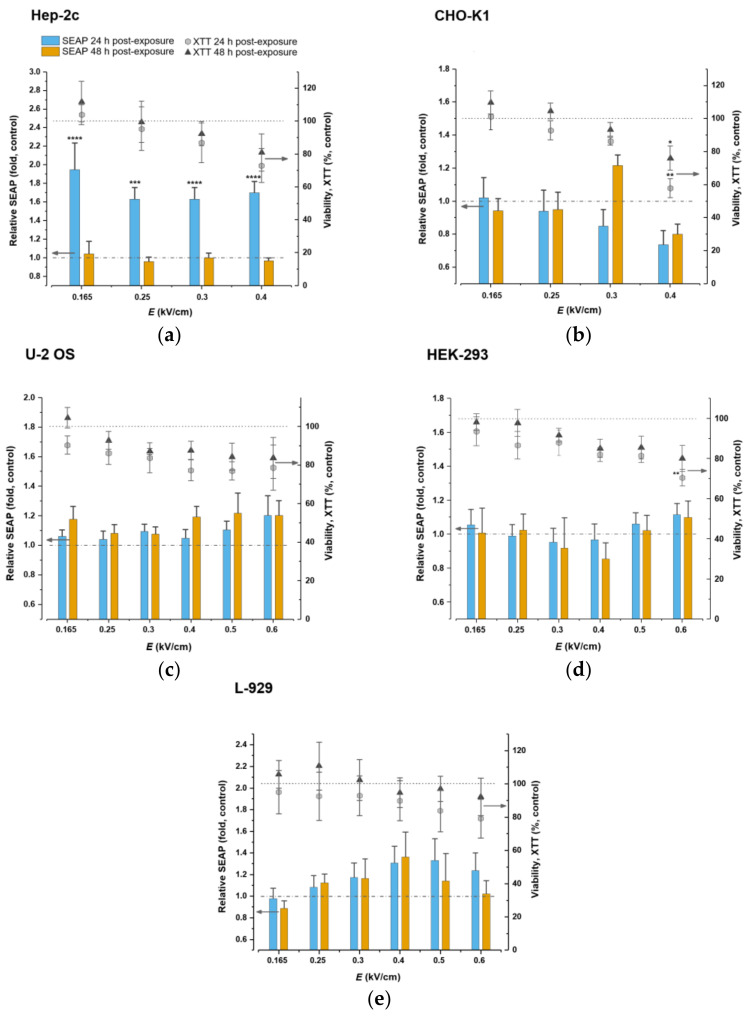
NF-κB/SEAP response system performance and viability readout in Hep-2c (**a**), CHO-K1 (**b**), U-2 OS (**c**), HEK-293 (**d**), and L-929 (**e**) cell lines after exposure to microsecond-duration pulsed electric fields. Hep-2c and CHO-K1 cells were stimulated with the electric field amplitudes of 0.165, 0.25, 0.3, and 0.4 kV/cm, eight pulses of 100 µs, frequency—1 Hz. U-2 OS, HEK-293, and L-929 cells were additionally simulated by applying the electric field amplitudes of 0.5 and 0.6 kV/cm. Results were read 24- and 48-h postexposure. Dashed lines denote baselines of negative SEAP and viability controls (cells cultivated under otherwise identical conditions, but in the absence of microsecond pulsed electric fields). Significances compared with control were determined using Kruskal–Wallis and Conover post hoc tests: * *p* < 0.05, ** *p* < 0.01, *** *p* < 0.001, **** *p* < 0.0001.

Membrane permeability study results revealed a direct correlation between the pulsed electric field strength and permeabilization rate in Hep-2c, U-2 OS, HEK-293, and L-929 cell lines. Differently, CHO-K1 cell membrane integrity remained intact or slightly permeable up to 0.5 kV/cm and was significantly affected only at 0.6 kV/cm (Figure 2a). In all cell lines, electric field strength of 0.165 kV/cm caused mild to no effect on membrane integrity. In Hep-2c, U-2 OS, HEK-293, and L-929 cells, permeability gradually increased starting from 0.25 kV/cm, and all cells, including CHO-K1, reached the highest membrane permeability point at 0.6 kV/cm—this also correlates with the reduced viability readouts that can be seen in Figure 3. Membrane reseal assay at 0.4 kV/cm (the point where most cells are significantly permeable but retain high viability levels) shows that membrane integrity loss is transient and returns to normal state after 300 s in nearly all cell lines (Figure 2b).

### 2.3. Cell Viability and NF-kB Pathway-Controlled SEAP Reporter System Response to Microsecond-Duration Pulsed Electric Fields Treatment

Pulsed electric field treatment was used to introduce changes in the expression levels of the NF-ĸB/SEAP reporter system. The electric field affects cell viability and may lead to lower cell survival rate. We examined the effects of different electric field strengths on cell viability. Obtained results demonstrate that viability of both Hep-2c and CHO-K1 cell lines decreased in a linear manner with increasing the electric field strength but remained high throughout 0.165–0.3 kV/cm 24- and 48-h postexposure (Figure 3a,b). However, in comparison with the untreated control (100%), viability declined by approximately 30% in Hep-2c (72.99% ± 10.39, *p* > 0.05) and 40% (57.83% ± 5.77, *p* < 0.01) in CHO-K1 24 h after the exposure to electric field strength of 0.4 kV/cm and recovered by approximately 20% 48 h postpulsation. Yet, higher electric field strengths deteriorated viability sharply 24- and 48-h postexposure (not shown); therefore, they were excluded from analysis in Hep-2c and CHO-K1 cells.

All pulsed electric fields strengths maintained high viability levels in U-2 OS, HEK-293 and L-929 cells at both time points (Figure 3c–e). However, 0.6 kV/cm reduced viability by approximately 30% in HEK-293 cell line (70.46% ± 4,17 p < 0.01) 24 h after application, yet it improved 24 h later and alleviated to approximately 80% compared with untreated control.

We further assessed the impact of microsecond-duration pulsed electric fields on the expression of the NF-kB pathway-controlled SEAP reporter system. After DNA transfection and delivery of electric pulses to Hep-2c cell line, we observed a significant stimulatory effect on the reporter system as SEAP levels boosted throughout 0.165–0.4 kV/cm 24 h postexposure (Figure 3a). The system attained highest activation at 0.165 kV/cm and its expression was approximately 2-folds (1.95 ± 0.29, *p* < 0.0001) higher compared with untreated control. SEAP levels, on average, were 1.6-fold higher at 0.25–0.4 kV/cm. The system settled 48 h postexposure since SEAP expression levels did not differ from the untreated control. The basal activity of the SEAP reporter system was low (not shown). Conversely, the system activity declined in CHO-K1 cells 24 h postexposure in a linear fashion while increasing electric fields strength (Figure 3b). SEAP levels were also similar 48 h after exposure, with one interesting exception—we observed a steady 1.2-fold (1.22 ± 0.06, *p* > 0.05) increase at 0.3 kV/cm. These differences, however, did not reach statistical significance. The basal activity of SEAP reporter system was high in CHO-K1 cells (not shown).

We also observed a slight stimulatory effect on the reporter system in U-2 OS cells at all electric fields strengths after both time points (Figure 3c) with several uncharacteristic peaks at 0.165, 0.4, 0.5, and 0.6 kV/cm 48 h postexposure. The highest 1.2-fold (1.22 ± 0.14, *p* > 0.05) system activity increase occurred at 0.5 kV/cm 48 h postexposure. Similarly, the reported system activity augmented slightly throughout 0.25–0.6 kV/cm in L-929 cells at both time points, with an exception at 0.6 kV/cm 48 h postexposure where the activity was equal to the control (Figure 3e). The highest peak occurred at 0.4 kV/cm and the SEAP levels mounted 1.4-fold (1.36 ± 0.23, *p* > 0.05). HEK-293 cells responded differently and, predominantly, remained unaffected by the treatment (Figure 3d). The SEAP activity lightly peaked at 0.6 kV/cm 24 h postexposure and resulted in a 1.1-fold (1.11 ± 0.06, *p* > 0.05) increase compared with the control. However, none of the differences observed in U-2 OS, HEK-293, and L-929 cell lines reached statistical significance.

## 3. Discussion

In this work, we described the experimental design and evaluation of a single gene response pattern to different parameters of microsecond-duration pulsed electric fields in five mammalian cell lines. It was a critical component in the study design to sensitively detect changes of the gene product; therefore, we used a nuclear factor kappa-B (NF-κB) promoter-controlled secreted embryonic alkaline phosphatase (SEAP) gene expression system for its physical stimulation responsive promoter. We also monitored viability and cell permeabilization readouts throughout the experiments. This is the first study that reveals microsecond-duration pulsed electric fields can alter gene expression and reinforces the concept that electronics can indeed manipulate genetics.

Previous observations that physical stimuli—UV light—can unleash NF-κB support our decision to use UV as an aid for the validation of the reporter system’s experimental setup [47,48,49]. We verified the setup by delivery of different irradiation doses to the cells. We found that UV stimulated the response system in Hep-2c, CHO-K1, U-2 OS, and HEK-293 cells, as SEAP levels significantly spurred in all of them. Cells of the subcutaneous connective tissue L-929, however, yielded different results—SEAP levels declined compared with the control. This demonstrated that the gene response to physical stimulation can be both positive and negative.

One of the most significant and exciting results was that the SEAP reporter gene expression significantly boosted in human cervix carcinoma Hep-2c cells in response to microsecond-duration pulsed electric fields. The effect was spotted 24 h postpulsation and resumed completely to the level of negative control after additional 24 h. An electric field amplitude of 0.165 kV/cm provoked the strongest 2-fold increase, but the effect persisted at higher amplitudes too. Interestingly, we observed that cell membrane integrity and viability were completely intact at 0.165 kV/cm. We knew from previous reports that pulsed electric fields of nanosecond-duration impact intracellular pathways, processes, and expression of different genes [39,43,50,51,52,53,54,55,56], but the fact that a similar effect can also be achieved by microsecond-duration pulsed electric fields is a new discovery. Microsecond-duration pulsed electric fields, meanwhile, were better known for their plasma membrane pore opening effects [57,58,59]. Low basal activity of the NF-κB-controlled system observed during our study in Hep-2c cells and its significant inducibility after pulsed electric fields application would allow to further analyze its use in primary cervical cancer cell cultures or even in an in situ setting. Similarly, reporter gene expression levels increased in other cancerous U-2 OS cells, but to a lower extent. The boost timing was different too—we could mainly detect stimulatory effects 48 h postexposure. As well as with Hep-2c, we detected the increase at 0.165 kV/cm and observed that cell membrane integrity and viability were altogether intact at this amplitude.

Previous attempts to regulate gene expression using long-millisecond-duration electric pulses achieved upregulation of the metallothionein I-controlled muSEAP reporter gene in mice connective tissue in vivo [29]. We checked if NF-κB-controlled SEAP expression would alter in mice connective tissue L-929 cells in response to microsecond-duration pulsed electric fields. Indeed, we observed a trivial trend towards upregulation of the reporter gene. The response intensity ascended and descended in a gradient manner: it topped at 0.4 kV/cm and persisted congruent at 24- and 48-h postexposure. At this amplitude, the viability remained high, yet permeability readings showed slight but significant damage to the cell membrane.

Ovarian tissue CHO-K1 cells yielded another set of interesting results. We knew from previous genomic studies that, in normal state, CHO-K1 upregulate nuclear factor κB and downregulate its inhibitor IκB as well as p53 [60]—the latter mutually inhibits NF-κB [61]. Indeed, during our study, we observed strong basal activity of the reporter system, but we also detected that its activity declined in response to microsecond-duration pulsed electric fields treatment (SEAP levels decreased in linear manner throughout 0.25–0.4 kV/cm 24 h postexposure), albeit slightly. One result stood out though—amplitude of 0.3 kV/cm caused upregulation of the NF-κB response system 48 h postexposure. Electric field amplitudes used for the response system study had no effect on cell membrane integrity whatsoever, but they caused cell toxicity as the viability deteriorated upon amplitude increase. It could be that a strong normal-state activation of the NF-κB interfered with pulsed electric fields and the effect failed to be clearly detected. Likewise, we observed a trivial trend towards downregulation in human embryonic kidney HEK-293 cells up to 0.4 kV/cm at each of the time points postexposure. It only averted at the highest amplitude 0.6 kV/cm, where we detected a slight stimulatory effect; however, at this point, we also observed cell toxicity as viability decreased significantly. Permeability readings showed damage to the cell membrane, and this was the only cell line that failed to reseal the membrane after 300 s. The slight stimulatory effect could have occurred due to increasing mechanical perturbations on the plasma membrane.

The main limitation of the study and reasons for triviality of our results on the levels of gene response could lie within the design of the study as it was solely tailored for screening of the effects of selected pulsed electric field parameters on different cell lines; therefore, the findings should be interpreted with caution. Nonetheless, our data allow us to draw preliminary conclusions that the NF-κB-controlled pathway can be potentially responsive to microsecond-duration pulsed electric fields, yet further studies specifically adjusting the parameters for different cell lines are necessary. Further, because of the short half-life of plasmids in the cell culture [62], we were unable to evaluate the behavior of the system after longer periods of time as well as its response to repeated exposures of pulsed electric fields. The present data from similar studies with millisecond-duration pulsed electric fields show that the response tends to fade over time and over the application of repeated impulses in vivo [29]; therefore, a stable cell line expressing NF-κB-controlled reporter protein could provide even more valuable insights on system behavior in the future studies. In addition, the studies of the low-frequency capacitively coupled electric fields (CCEF), used for the regeneration of bone tissue, demonstrated the induction of the chromosomal alkaline phosphatases (ALP) 4 h postpulsation in human bone SaOS-2 cell line, where the effect resided fully 24 h postpulsation [63]. Differentially modulated mRNAs were also detected 24 h after exposure to CCEF in SaOS-2 cells [64]. The stable cell line expressing NF-κB pathway-controlled reporter protein could be used in a similar study for direct comparison of expression of native ALP versus modified SEAP after the application of diverse nature physical stimuli in a time-dependent manner and confirm if the system could be used as an artificial switch if different SEAP levels are demonstrated.

We hypothesize that reactive oxygen species (ROS) play a crucial role in either positive or negative regulation of the NF-κB signaling pathway after delivery of electric pulses because ROS can lead to both inhibitory and stimulatory responses [65]. The delivery of high-field electric pulses to mammalian cells can generate free radicals [66,67]. The quantity of highly reactive oxygen species depends on the parameters of the electric pulses applied to cells (field intensity, frequency, number and duration of the pulses, cell concentration). The generation of ROS after exposure to pulsed electric fields can be direct when free radicals form at the surface of the electrodes and indirect when free radicals form inside the cells in response to electrical stimulation [68]. We speculate that another mechanism could involve regulation by calcium ions—a preceding research revealed that microsecond-duration pulsed electric fields can permeabilize the endoplasmic reticulum membrane of mammalian cells, causing the leak of Ca^2+^ [69]. Fluctuation of intracellular Ca^2+^ levels, in turn, might modulate NF-κB activity [70,71,72]. Similarly, millisecond-duration electric pulse application upregulated the NFAT-promoter-controlled SEAP gene expression system in HEK-293T cells; however, this required additional co-transfection with plasmids encoding the L-type voltage-gated calcium channels [27]. Lastly, the research of pulsed electric fields’ impact on cell plasma membrane fluidity and state of cytoskeleton, support the idea of microsecond-duration pulsed electric fields as an abiotic factor triggering the NF-κB pathway [73]. NF-κB acts as a sensor of actin reorganization due to mechanical affliction on the plasma membrane [74].

The possibility to control gene expression using mild biophysical stimulation, concomitantly with other physical techniques, would be a significant improvement over the use of small molecules, such as steroids or antibiotics. The present study, for the first time, proves the principle that microsecond-duration pulsed electric fields, under certain parameters, can alter single-gene expression in plasmid context in various cell lines without causing significant damage to cell integrity or viability. The gene expression might either be upregulated or downregulated depending on the cell line and parameters applied. This noninvasive, ligand-, cofactor-, and nanoparticle-free approach enables easily controlled, direct electrostimulation of the construct carrying the gene of interest; the discovery may contribute towards the path of simplification of the complexity of physical systems used for gene regulation and provide a solution to the downsides of exogenous small molecules. Given the advantages of microsecond-duration pulsed electric fields technology and our findings on modulation of the transcription-based gene control system, future studies evaluating specific parameters on different cell lines, including primary cells as well as different transcription systems—for example, stable transcription systems or systems, reduced to their minimal parts—would shed more light before the approach can be endorsed for gene therapy purposes.

## 4. Materials and Methods

### 4.1. Experimental Setup

**Figure 4:**
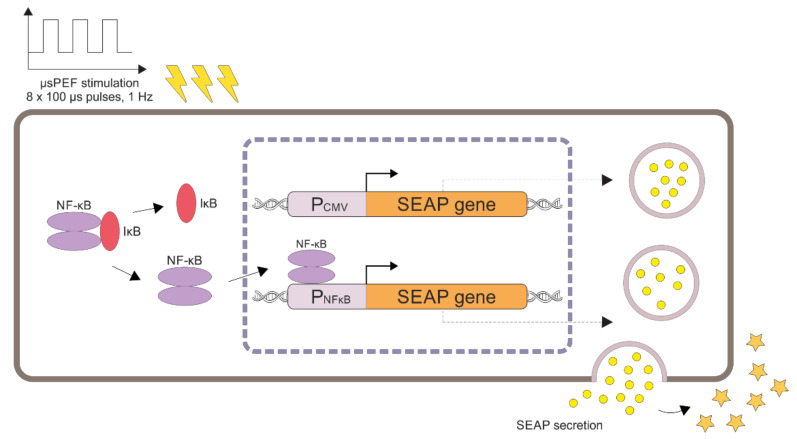
Hypothetical schematic representation of electrically modulated transcription of the SEAP reporter gene. Pulsed electric fields activate the nuclear factor κB pathway, leading to its release from the inhibitor and translocation to the nucleus. NF-κB binds to a synthetic promoter and activates the transcription of the SEAP reporter gene. The SEAP protein secretes to the cell growth medium and converts the substrate (p-nitrophenyl phosphate, PNPP) into a yellow detectable product.

To monitor physical-stimuli-induced quantitative changes of secreted embryonic alkaline phosphatase (SEAP) expression, we developed a detection system consisting of Hep-2c, U-2 OS, HEK-293, CHO-K1, and L-929 cells transfected with the inducible expression vector pNF-κB-SEAP. The vector bears the P_NFĸB_ promoter that drives transcription of the SEAP reporter gene in response to the activation of the NF-κB pathway. SEAP protein secretes into the growth medium of cells, where it can be detected by conducting an enzymatic reaction (using p-nitrophenyl phosphate (PNPP) substrate that develops a yellow hue upon reaction with the alkaline phosphatase) followed by a colorimetric analysis (Figure 4). Growth medium from cells cultivated under otherwise identical conditions, but in the absence of the physical stimulus, served as negative control. Cells transfected with the pCMV-SEAP constitutive expression plasmid (that bears the P_CMV_ constitutive promoter) indicated a successful transfection in every experiment (not shown).

### 4.2. Cell Culture

Human cervix carcinoma Hep-2c cells (Merck, Darmstadt, Germany) were cultured in the RPMI 1640 medium (Corning Inc., Corning, NY, USA). Chinese hamster ovary CHO-K1 cells (ATCC^®^ CCL-61™; American Type Culture Collection, Manassas, VA, USA) were cultured in Ham’s F-12K Nutrient Mixture (Corning Inc., Corning, NY, USA). Human bone osteosarcoma U 2 OS cells (ATCC^®^ HTB 96™; American Type Culture Collection, Manassas, VA, USA), human embryonic kidney 293 [HEK-293] cells (ATCC^®^ CRL1573™; American Type Culture Collection, Manassas, VA, USA; referred to as cell line HEK-293), and mouse subcutaneous connective tissue NCTC clone 929 [L cell, L-929, derivative of Strain L] cells (ATCC^®^ CCL-1™; American Type Culture Collection, Manassas, VA, USA; referred to as cell line L-929) were cultured in the Dulbecco’s Modified Eagle Medium (DMEM; Corning Inc., Corning, NY, USA). The growth medium was supplemented with a 10% fetal bovine serum (FBS; Fisher Scientific, Waltham, MA, USA) for all cell lines. Cells were grown in T25 cell culture flasks (Fisher Scientific, Waltham, MA, USA) as a monolayer culture and routinely passaged after 90–100% confluency was reached. Cell cultures were maintained at 37 °C under a 5% CO_2_, 95% air atmosphere.

### 4.3. Plasmids

Two SV40-based plasmids were used in this study (Imgenex, San Diego, CA, USA). The pNF-κB-SEAP plasmid carries the placental secreted alkaline phosphatase (SEAP) reporter gene under the control of inducible ELAM promoter containing nuclear factor ĸB recognition sites (P_NFĸB_). The recombinant form of alkaline phosphatase, designed by inserting a translational terminator after the amino acid 489, functions as the reporter gene [75]. This mutation converts native membrane-bound protein into a secreted form. The pCMV-SEAP plasmid carries the SEAP reporter gene under the control of the constitutive cytomegalovirus promoter (P_CMV_), used as positive control for transfection efficiency, and SEAP colorimetric reaction (not shown).

### 4.4. Plasmid DNA Transfection

For plasmid DNA transfection, the cell lines were propagated until 90% confluence was reached and harvested using a 0.25% trypsin (Sigma-Aldrich, St. Louis, MO, USA), 0.53 mM EDTA (Carl-Roth, Karlsruhe, Germany) solution. Cells were counted using a 0.100 mm/0.0025 mm^2^ Neubauer hemocytometer (Hecht Assistent, Sondheim vor der Rhön, Germany) and transferred to 96-well cell culture plates (Corning Inc., Corning, NY, USA) at a cell density of 1.5 × 10^4^ cells per well. All cell lines were transfected with plasmid DNA once 70% confluence was reached, using a Lipofectamine 3000™ Transfection Kit (Fisher Scientific, Waltham, MA, USA) according to the manufacturer’s protocol.

### 4.5. Cell Exposure to Ultraviolet Light

Ultraviolet (UV) light stimulation was applied to all cell lines 24 h posttransfection in each respective growth medium. Cells were plated into 96-well culture plates and exposed to UV light (365 nm, 60 W) in the UV-Exposure Box 2 (Proma Systro, Eiterfeld, Germany). During experiments, the UV dosage was calculated according to Equation (1). Here, *D* represents the applied UV dosage, *I* is irradiance, and *t* is time of exposure.

(1) $$D\left[\frac{J}{{\mathrm{cm}}^{2}}\right]=I\;\left[\frac{W}{{\mathrm{cm}}^{2}}\right]\cdot t\left[s\right]$$

The samples were exposed to 4.14 J/cm^2^ and 20.7 J/cm^2^ irradiation doses at a path length of 1 cm. For the L-929 cell line, an additional UV irradiation dose of 41.7 J/cm^2^ was applied. Cells without exposure to UV, harboring the pNF-κB-SEAP plasmid, were used as negative control. Cells transfected with the pCMV-SEAP plasmid were used as positive control for transfection efficiency and SEAP colorimetric reaction. Cell culture media samples were taken 24 h after exposure for reporter gene expression evaluation. Every experiment was conducted in duplicate and repeated at least 3 times for each exposure setting. Each independent experiment was conducted on different days with freshly prepared cell cultures.

### 4.6. Cell Exposure to Microsecond-Duration Pulsed Electric Fields

Pulsed electric field stimulation was applied to all cell lines 24 h post plasmid transfection. Cells were washed twice with phosphate buffered saline (PBS; Medicago, Berga, Denmark), harvested with trypsin/EDTA solution, and centrifuged at 400× *g* for 5 min. Further, the pellet was resuspended in 50 µL of Hank’s Balanced Salt solution (HBSS; Fisher Scientific, Waltham, MA, USA) and transferred to an electroporation cuvette. Pulsed electric field was applied using the high-power square wave pulse electroporator Elpora (developed at the Centre for Physical Sciences and Technology, Vilnius, Lithuania [30]). The spacing between the cuvette (Fisher Scientific, Waltham, MA, USA) electrodes was 2 mm. During experiments, the strength of the electric field was calculated according to Equation (2).

(2) $$E\left[\frac{\mathrm{kV}}{\mathrm{cm}}\right]=\frac{U_{CUV}\left[V\right]}{d\left[\mathrm{mm}\right]}\cdot 100$$

Here, *U_cuv_* is the voltage drop across the cuvette electrodes and *d* is the distance between electrodes. The voltage across electrodes and pulse shape were measured using an oscilloscope embedded into the Elpora device. A train of eight pulses with duration of 100 µs and repetition frequency of 1 Hz was applied. The applied voltages resulted in electric field amplitudes of approximately 0.165 kV/cm, 0.25 kV/cm, 0.3 kV/cm, 0.4 kV/cm, 0.5 kV/cm, and 0.6 kV/cm, respectively. Cells without exposure to microsecond-duration pulsed electric fields, harboring the pNF κB SEAP plasmid, were used as negative control. Cells transfected with the pCMV-SEAP plasmid were used as positive control for transfection efficiency and SEAP colorimetric reaction (not shown). Cell culture media samples were taken 24- and 48-h after microsecond-duration pulsed electric fields’ exposure for the reporter gene expression evaluation. Every experiment was carried out in duplicate and repeated not less than 5 times for each microsecond-duration pulsed electric fields exposure setting. Each independent experiment was conducted on different days with freshly prepared cell cultures.

### 4.7. SEAP Reporter Gene Expression Evaluation

The activity of the reporter gene was analyzed using SEAPorter™ Assay Kit (Imgenex, San Diego, CA, USA) according to the manufacturer’s instructions using cell growth medium samples, diluted at a ratio of 1:2. Background endogenous alkaline phosphatases were inactivated by preincubation at 65 °C for 30 min. Inactivated samples were transferred into a 96-well plate, and levels of SEAP were determined by adding 50 µL of 2 mg/mL PNPP (Fisher Scientific, Waltham, MA, USA) substrate solution into each well. Samples were incubated at room temperature for 60 min and SEAP levels were measured using the Sunrise™ microplate absorbance reader (TECAN, Grödig, Austria) at a wavelength of 405 nm. The absorbance of the blank control wells was subtracted from the test samples. SEAP protein concentration (ng/mL) was calculated based on the SEAP standard curves. Data are presented as fold comparisons of the concentrations of SEAP per activity after microsecond-duration pulsed electric fields or UV exposure to negative control.

### 4.8. Metabolic Activity (Viability) Assay

Cellular metabolic activity was evaluated after microsecond-duration pulsed electric fields treatment by the XTT (2,3-bis-(2-methoxy-4-nitro-5-sulfophenyl)-2H-tetrazolium-5-carboxanilide) assay, using the Cell Proliferation Kit (Biological industries, Beit-Haemek, Israel) according to the manufacturer’s protocol. The cellular metabolic activity was assessed 24- and 48-h postpulsation. The growth medium was removed and 100 µL of new appropriate medium was added. Then, cells were incubated with 50 µL of XTT solution in 96-well cell culture plates at 37 °C in a 5% CO_2_ chamber for 2 h. After incubation, the formed formazan dye was quantitated at 450 nm using a multiwell plate reader, Sunrise™. Every experiment was conducted in duplicate. The absorbance of the blank control wells was subtracted from the test samples. Cell count was calculated based on the XTT absorption standard curves. Cellular metabolic activity (viability) is presented as a percentage comparison to the untreated negative control cells.

### 4.9. Membrane Permeabilization Assay

Membrane permeabilization was evaluated for all cell lines and all microsecond-duration pulsed electric field parameters used in this study. A membrane impermeable nucleic acid stain SYTOX^®^-green (Invitrogen, Carlsbad, CA, USA) was used for analysis. Cell lines were propagated in cell culture flasks until 85–90% confluency, washed twice with PBS, detached with trypsin/EDTA, and harvested by centrifugation. The cells were counted, resuspended in HBSS at cell density of 2 × 10^4^, and transferred to cuvettes for microsecond-duration pulsed electric fields treatment. Immediately after exposure to microsecond-duration pulsed electric fields, samples were moved to black fluorescence measurement microplates (Fisher Scientific, Waltham, MA, USA) and 50 μL of 2-µM SYTOX^®^-green solution was quickly added to each well. Fluorescence signals were measured one hour later with a Clariostar Plus fluorimeter (BMG Labtech, Ortenberg, Germany), with excitation and emission at 485 nm and 523 nm, respectively. Every measurement was conducted in duplicate and repeated at least two times. Readings of untreated cells with and without added stain were regarded as negative control. Results are presented as fold comparisons of the fluorescence of SYTOX^®^-green after microsecond-duration pulsed electric fields to negative control.

### 4.10. Data Analysis

Data are reported as mean ± standard error (SE) values. Kruskal–Wallis and Conover post hoc tests were carried out to evaluate SEAP and viability after pulsed electric fields exposure, whereas pairwise Student’s *t*-test was applied to evaluate SEAP after UV exposure, cell permeability, and recovery assays. The nonparametric test (Kruskal–Wallis) was chosen because, in our view, the data disobey the assumption for normal distribution. Statistical analyses were conducted using R (R Core Team (2021)). *p* values less than 0.05 indicated statistical significance.

## Data Availability

The data presented in this study are available on request from the corresponding author.
